# Table tennis and cognitive skills: A study on attention and decision making in sports science students

**DOI:** 10.1371/journal.pone.0353175

**Published:** 2026-07-08

**Authors:** Nurullah Çelik, Ceren Bolat, Ahmet Dönmez

**Affiliations:** 1 Sakarya University of Applied Sciences, Faculty of Sport Sciences, Sakarya, Türkiye; 2 Independent Researcher, İstanbul, Türkiye; 3 Alanya Alaaddin Keykubat University, Faculty of Sport Sciences, Antalya, Türkiye; İzmir Democracy University: Izmir Demokrasi Universitesi, TÜRKIYE

## Abstract

This research aimed to reveal the effects of table tennis on students’ careful decision-making and concentration skills. The study employed a quasi-experimental pretest–posttest control group design with groups assigned through random allocation. The study was carried out with a total of 28 students (X_age_ = 20.65 ± 1.70), 14 of whom were studying at the department of physical education and sports of a state university and taking an elective table tennis course (X_age_ 19.79 ± 1.89) and 14 who did not take a table tennis course (X_age_ 21.43 ± 1.16). The data in the research were obtained using the “Personal Information Form”, “D2 Attention Test” and “Melbourne Decision Making Scale”. Analysis of variance (2x2 ANOVA) was used for repeated measures in the analysis of data. According to the research findings, an increase was observed in the pre-test–post-test mean scores for concentration and careful decision-making in the experimental group. Similarly, a smaller increase was observed in the control group. Analysis results revealed that the time effect was significant for the variables of concentration performance (F = 16.328; p < .001) and careful decision-making (F = 4.759; p = .038). However, it was determined that the group × time interaction was not significant for the variables of concentration performance (F = 1.879; p = 0.182) and careful decision-making (F = 3.933; p = 0.058). In conclusion, while table tennis is thought to contribute to the development of attention and decision-making skills, further studies with larger sample sizes are needed to more clearly establish the intervention’s effects.

## 1. Introductıon

Table tennis is often considered a reaction-based sport due to the high speed of the ball and the short distance between players [1]. When table tennis players make a physical countermove by predicting the opponent’s stroke, they not only watch the opponent’s stroke, but also observe the contact of the ball and the racket, and similar situations. Once there is visual certainty with the contact of the racket and the ball, the previously acquired intuition-related information allows the move to be predicted. At this time, it is expected from the biomechanics of the movement, the sound that comes with the ball’s bounce [2]. Thus it can be said that attention skills need to be developed, or that the movements applied while predicting the opponent’s movements in table tennis improve attention and decision-making while thinking about how to stroke. Jeoung [3] states that table tennis is more effective for cognitive function compared to other branches. While it physically develops joints and muscles, hand-eye coordination, and agility, it is thought that it affects skills such as decision making, concentration, attention, estimation, and analytical thinking since it progresses cognitively by establishing strategies. Similar findings regarding the positive effects of movement-based cognitive exercises on attention and cognitive performance have also been reported in recent studies [4].

Today, decision-making is considered important in management science in theory and managerial processes in practice. Decision Theory is grouped as Classical, Rational, and Limited Rational Decision Theory. Classical Rational Decision Theory states that the person making the decision acts according to all possibilities and consequences. Herbert Alexander Simon, who criticized this theory, accepted the concepts of decision making and “management” as the same in Limited Rational Decision Theory (1965) and stated that it cannot be rational and that the person makes a managerial decision according to reality and limitations. He said that a person who progresses towards his goal chooses the decision that satisfies him when faced with various paths [5]. To give an example from the relationship of this theory with sports branches, it can be associated with the decision of a person to make a move by choosing the most suitable path for himself while playing table tennis. In addition to the cognitive functions that table tennis develops, it can be said that table tennis is one of the most preferred elective courses chosen by students at universities because it is an entertaining sport. Previous studies conducted in different sports disciplines have also emphasized the contribution of sports participation to cognitive flexibility, problem-solving, and behavioral regulation skills [6]. It is thought that the development of students’ concentration and careful decision-making skills in table tennis lessons, which are preferred consciously or unconsciously in the development process, can also affect their daily and academic lives. In this context, psychological factors associated with sports participation may also contribute to cognitive processes and behavioral responses during performance [7]. If we associate the table tennis lesson with the three stages (cognitive, association, autonomy) suggested by Fitts & Posner [8] for skill learning; in the cognitive stage, which is the first learning stage, where decision-making processes take place and attention need is high, the development of the skills gained in table tennis at the beginning of the lesson is low and the attention need for it is high. In the last stage, namely autonomy, movements become automatic, careful decision-making and concentration skills are acquired, and the attention need for it is less.

When the studies are examined, it is seen that information technology should be applied to encourage learning in table tennis education applied in universities [9], table tennis skill outcomes differ when different teaching methods are used [10], when table tennis athletes and ordinary university students are compared, attention control is at a higher level in the table tennis group [11], table tennis players exhibit superior visual attention, which contributes to better foresight and decision-making during the game. Regarding the [12] effect of table tennis, it is seen that recent studies have mainly focused on the development of physical skills such as body composition [13–15] and mental health issues [16,17]. In addition, it has been stated that table tennis has a positive effect on attention deficit and hyperactivity disorder [18]. Since there are limited studies that include the effect of table tennis on attention skills such as careful decision-making and concentration, for university students, this study aimed to contribute to the literature and to test the cognitive features developed by the table tennis course. In this context, the present study specifically aims to fill the gap in the literature regarding the combined evaluation of attention-related cognitive skills such as careful decision-making and concentration within the scope of table tennis education at the university level. Unlike previous studies that predominantly focus on physical outcomes or general cognitive benefits, this study adopts a more focused approach by examining specific attention components. Methodologically, the study differs from earlier research by addressing these variables together within an educational setting. Additionally, the characteristics of the sample, consisting of university students who have selected table tennis as an elective course, provide a distinct perspective in understanding how structured sports education influences cognitive development. Therefore, the findings are expected to contribute to the literature by offering a more detailed understanding of the role of table tennis in the development of attention-based cognitive skills. In addition, the variables examined in this study—careful decision-making and concentration—are considered core components of attention in the literature, yet their combined assessment within the context of table tennis education remains limited. Therefore, this study provides a more specific and integrative contribution to the field.

## 2. Method

### 2.1. Research model

The study was conducted through a pre-test post-test model with a control group consisting of groups determined by unbiased assignment, which is one of the quasi-experimental research methods. According to Fraenkel, Wallen and Hyun [19], conducting an experimental research is only possible by providing all treatments to a single subject. In this case, all members of the experimental group are subjected to the same treatment, while the control group is not treated at all.

### 2.2. Research group

The study consisted of 28 students enrolled in the Department of Physical Education and Sports at a state university. The sample included 10 female and 18 male students. Participants were divided into an experimental group consisting of 14 students who attended the table tennis course (M_age_ = 19.79 ± 1.89 years) and a control group consisting of 14 students who did not attend the course (M_age_ = 21.43 ± 1.16 years). The overall mean age of the participants was 20.65 ± 1.70 years. Participants were assigned to groups through random allocation. All participants were physically active university students and voluntarily agreed to participate in the study.

Table 1 shows the descriptive characteristics of the participants included in the study. A total of 28 university students participated in the research, consisting of 10 females (35.7%) and 18 males (64.3%). Regarding group distribution, 14 participants (50.0%) were assigned to the experimental group and 14 participants (50.0%) to the control group. These findings indicate that the groups were equally distributed in terms of participant numbers.

**Table 1:** Descriptive characteristics of the participants.

Variable	Category	n	%
Gender	Female	10	35.7
	Male	18	64.3
Group Type	Experimental	14	50.0
	Control	14	50.0
Total		28	100.0

#### 2.2.1. Sample size and power analysis.

The sample size was determined using G*Power 3.1 software [20]. An a priori power analysis was conducted for repeated measures ANOVA with a within–between interaction design. Based on Cohen’s [21] recommendations, a medium effect size (f = 0.25), an alpha level of 0.05, and a statistical power of 0.90 were specified. The analysis indicated that a minimum sample size of 30 participants was required. Although the present study included 28 participants, the sample size was considered acceptable given the practical limitations and the exploratory nature of the study.

### 2.3. Data collection tools

Data were collected using the “Personal Information Form” containing the students’ age and gender characteristics, the “D2 Attention Test” to measure the students’ attention levels, and the “Melbourne Decision Making Scale” to determine the students’ decision-making skills.

#### 2.3.1. Personal information form.

The personal information form prepared by the researchers consists of questions aimed at learning personal information, such as age and gender of the students participating in the study.

#### 2.3.2. Melbourne decision making questionnaire (DMQ).

In the study, the ‘’Melbourne Decision Making Questionnaire,” which was developed by Mann et al. [22] and adapted to Turkish by Deniz [23] was used to determine the decision-making skills of the students. The scale consists of 2 parts. Part I: It aims to determine self-esteem in decision-making. It consists of six items, three of which are scored directly, and three items are scored in reverse. Scoring is done as follows: “True” response to 24 items is 2 points, “Sometimes True” response is 1 point, “Not True” response is 0 points. The maximum score that can be obtained from the scale is 12. High scores indicate high self-esteem in decision-making. Part II: It consists of 22 items and measures decision-making styles. There are 4 sub-dimensions for different decision-making styles in the scale, and the internal consistency coefficients of the scale sub-dimensions vary between.65 and.80 [23]. Reliability analyses were conducted to assess the internal consistency of the measurement instruments used in the study. The Cronbach’s alpha coefficient for the careful decision-making subscale of the Melbourne Decision Making Scale was found to be α = .781 for the pre-test and α = .757 for the post-test. These findings indicate an acceptable level of internal consistency reliability for the measurement tool used in the study [24].

#### 2.3.3. D2 attention test.

In the study, the ‘’D2 Attention Test” developed by Brickenkamp [25] was used to evaluate the sustained attention and visual scanning skills of the students. This test measures selective attention and mental concentration. Çağlar and Koruç [26] reported that the validity and reliability of the D2 attention test, which measures attention in terms of performance, were high and could be used in research and application areas in the study. A validity-reliability study was conducted on Turkish athletes aged 12 and over. A total of 701 athletes, 437 male and 264 female, from various sports branches (athletics, basketball, gymnastics, football, weightlifting, handball, archery, wrestling, volleyball, sailing and swimming) from a total of 5 age groups, 12–15, 16–20, 21–24, 25 and above, were studied. Cronbach’s alpha internal consistency coefficient was evaluated to obtain internal consistency coefficients, and internal consistency coefficients of all subgroups were found to be above 0.70. The test includes an area for writing personal information and performance results for recording purposes, and a test line. Each line contains 47 letters and a total of 14 lines. Each line contains 16 different signs, including the letters p and d, which are small commas between 1 and 4. In the test, the individual scans to find the letter d with two signs and crosses them out, without evaluating the incorrect letters in the line. The test was applied as a group in a classroom environment, and 20 seconds were given for each line. The total item in the evaluation is the performance measurement for all the items marked correctly or incorrectly. Error includes the sum of the unmarked correct items and the incorrectly marked ones. Percentage error is a parameter that measures performance qualitatively. It is the error rate made in all the selected items. As the error rate decreases, accuracy, quality, and attention performance of the individual increase. It is found by the ratio of error to the total item. Marked correct items are found by subtracting the total error score from the total number of scanned items and give the number of correct items. Concentration performance is obtained by subtracting the incorrectly marked items from the number of correctly marked items. Only concentration scores were used for the purpose of this research.

### 2.4. Procedure

Before starting the research, permission was obtained from the Sakarya University of Applied Sciences Ethics Committee with the number E-26428519-050.99-112630 and dated 11/01/2024. At the beginning of the course, information about the study was given to the students who would participate in the study and the consent forms were read and signed. Then, in order to determine the groups, scales were applied to each student and pre-test scores were obtained.

After the statistical process, the selection of the groups was made by unbiased assignment. After the pre-tests were taken, the students who took table tennis lessons were included in the experimental group. The students in this group were taught serve, forehand, and backhand stroke techniques, forehand, and backhand return skills during 8 weeks. Serve technique was practiced in the first 2 weeks, forehand stroke technique in the 3rd and 4th weeks, backhand stroke technique in the 5th and 6th weeks, and forehand and backhand return techniques in the 7th and 8th weeks. Each technique consisted of 3 sessions of 30 minutes. All training sessions were supervised and conducted by the first researcher, who had experience in table tennis instruction. In addition to technical training, participants performed ball tracking, hand–eye coordination, reaction, and stroke control exercises throughout the intervention period. Furthermore, the intensity and complexity of the exercises were progressively increased according to the participants’ skill development during the intervention process. The control group did not receive any training and continued their regular educational activities during the intervention period.

After the application, scales were applied to the control and experimental groups for the pre-test and post-test. At the end of the eight weeks, the same scales were applied to the experimental and control groups again, post-test data were obtained, and the research was concluded. The intervention period was conducted between March 4, 2024, and April 26, 2024.

### 2.5. Data analysis

The data obtained from the individuals participating in the study were numerically coded and transferred to the SPSS 25.0 statistics program. The conformity of the data to normal distribution was evaluated by examining the skewness and kurtosis values. As a result of the analysis, it was determined that these values were between −2 and +2. There is evidence in the literature that the values remaining in this range meet the normal distribution assumption [27]. In the analysis of the data, the repeated measures ANOVA method was used for repeated measurements and the statistical significance level was accepted as p < .05. In addition, the percentage change rates depending on time were calculated using the formula “%Δ= (Posttest–Pretest)/Pretest*100” [28].

## 3. Findings

Table 2 presents the results of the analysis conducted to determine the differences between the experimental and control groups, according to the groups and based on the pre-test and post-test concentration values.

**Table 2:** Comparison of CP values of participants according to groups and measurement times.

Variables	n	Pre-test	Post-test	Total	%Δ	F	p
		$\overset{―}{𝐗}$ ± SS	$\overset{―}{𝐗}$ ± SS	$\overset{―}{𝐗}$ ± SS			
Experiment	14	155,71 ± 29,42	177,43 ± 23,24	166,57 ± 26,33	%12,65	4,077	,054
Control	14	139,07 ± 32,72	149,79 ± 36,63	144,43 ± 34,68	%7,70		
Total	28	147,39 ± 31,69	163,61 ± 33,23				
		F = 16,328; p = ,000			Group X time interaction F = 1,879; p = ,182		

According to the Table 2, it was determined that the CP values of the participants did not differ according to the experimental and control groups (F = 4.077; p = .054). It was determined that the pre-measurement and post-measurement averages of the participants differed according to time (F = 16.328; p = .000). According to this result, while there was a 12.65% increase in the values of the experimental group, there was a 7.70% increase in the values of the control group. Finally, no significant difference was obtained in the group-time interaction (F = 1.879; p = .182).

Table 3 presents the results of the analysis conducted to determine the differences between the experimental and control groups, according to the groups and based on the pre-test and post-test careful decision-making scores.

**Table 3:** Comparison of participants’ careful decision-making values according to groups and measurement times.

Variables	n	Pre-test	Post-test	Total	%Δ	F	p
		$\overset{―}{𝐗}$ ± SS	$\overset{―}{𝐗}$ ± SS	$\overset{―}{𝐗}$ ± SS			
Experiment	14	7,00 ± 1,04	8,50 ± 2,24	7,75 ± 1,64	%21,43	,876	,358
Control	14	7,29 ± 1,44	7,36 ± 1,15	7,32 ± 1,30	%0,96		
Total	28	7,14 ± 1,24	7,93 ± 1,84				
		F = 4,759; p = ,038			Group X time interaction F = 3,933; p = ,058		

According to Table 3, it was determined that the careful decision-making values of the participants did not differ according to the experimental and control groups (F = ,876; p = ,358). It was determined that the pre-measurement and post-measurement averages of the participants differed according to time (F = 4.759; p = ,038). According to this result, while there was a 21.43% increase in the values of the experimental group, there was a 0.96% increase in the values of the control group. Finally, no significant difference was obtained in the group-time interaction (F = 3.933; p = ,058).

## 4. Dıscussıon and conclusıon

This study investigated the effects of eight weeks of table tennis on the concentration and careful decision-making skills of sports science students. According to the findings of the study, while the concentration scores of the participants did not differ according to the groups, the pre-test and post-test measurements differed according to time, and it was observed that the concentration scores of the students who took table tennis lessons increased by 12.65% and the concentration scores of the students who did not take table tennis lessons increased by 7.70%. The observed improvements in both groups may be partially explained by a test familiarity effect, as participants completed the attention test for a second time at post-measurement and may have demonstrated improved performance due to increased familiarity with the test format and requirements. Nevertheless, the greater numerical increase in the table tennis group suggests that table tennis training may have contributed to improved concentration performance.

Regarding the careful decision-making sub-dimension, no statistically significant differences were found between groups; however, a significant time effect was observed between pre-test and post-test measurements. While the table tennis group showed a 21.43% increase, the control group exhibited only a marginal increase of 0.96%. Although the group-by-time interaction was not statistically significant, the greater increase observed in the table tennis group may suggest a potential positive effect of table tennis training on careful decision-making skills; however, this interpretation should be considered cautiously due to the absence of statistically significant between-group differences.

Table tennis affects cognitive skills as well as physical skills. Since players have to observe and think about the opponent and the ball in addition to their own moves, it can be said that careful decision-making skills and concentration skills are developed by monitoring the opponent’s movements and planning to shoot at the targeted place by ensuring ball control. Similar to the attention test used in this study in the literature, the Kana Pick-out test was applied to table tennis players to measure attention and attention division skills [29,30]. In the study conducted by Kawano, Mimura, and Kaneko [29] on adults aged 50 and over, it was determined that participants who played table tennis received higher scores than participants who did not play. Mori and Sato [30] applied the Kana Pick-out Test on participants aged 10–70 and obtained similar results. In an electroencephalographic study (a method that measures electrical monitoring of brain wave activity) conducted on adults, it was observed that cognitive tasks were performed better while playing table tennis compared to some branches such as cycling, and therefore table tennis developed brain regions related to motor control, attention processing, decision-making and executive function more effectively [17,31]. Zhang, Chen, Wu, and Li [12] stated that mental training interventions in table tennis improved players’ accuracy, consistency, and decision-making abilities during the match; cognitive functions such as reaction time, attention, and working memory played an important role in executing complex motor actions and making effective decisions during the game. Huang, Xuechen, Jilong, Jun, and Anmin [32] found that table tennis athletes were superior in controlling behavior, attention, and emotional state in the context of both domain-general and domain-specific tasks. As a result of a study conducted with handball, basketball and table tennis players by applying tests such as reaction time, agility, sprint, and squat, it was revealed that the performance of table tennis players is determined by the speed at which they respond to visual stimuli [33]. Haryanto and Amra [34] determined that concentration and hand-eye coordination have a strong enough relationship with backhand backspin serve by applying a grid test for concentration and a tennis ball throwing test for hand-eye coordination. However, they stated that concentration and eye-hand coordination should be trained to increase the athlete’s serve accuracy while giving table tennis training. In another study, Chen, Chueh, and Hung [35] revealed that table tennis players, regardless of their proficiency levels, exhibited more advanced processing speed in visual-spatial tasks than non-athletes and that their attention and memory performances were more advanced. In an experimental study aimed at investigating the effect of table tennis training programmed with studies focused on mental development on improving the cognitive abilities of beginner-level tennis players, it was concluded that athletes could effectively improve their cognitive abilities by using a different attention test from this study [36]. When examining the duration of the studies, it is considered that the 8-week period of the study may be insufficient, as training was conducted over periods of 8 months [13] and 2 years [15]. The findings reported in the literature support the observation that students who received table tennis instruction achieved higher scores than those who did not across all analyses conducted in this study. However, prior studies have clearly demonstrated the cognitive effects of table tennis, which appears to contrast with the present study’s finding of no significant group × time interaction. This discrepancy may be attributed to differences in the sample characteristics and the implementation processes employed across studies.

When the obtained results are evaluated, it is observed that there is no statistically significant Group × Time interaction for either concentration or careful decision-making variables. Although the improvement observed in the table tennis group appears more pronounced compared to the control group, these changes cannot be conclusively attributed to the intervention effect. Therefore, the observed differences should be interpreted as descriptive trends rather than evidence of a causal relationship. Although the Group × Time interaction did not reach statistical significance in terms of variables, the higher rate of improvement observed in the table tennis group compared to the control group may indicate a potential beneficial effect of table tennis training. However, since the observed improvements could also be explained by repeated testing, familiarity with measurement procedures, and natural changes over time, these findings should be interpreted with caution. Furthermore, the interaction effect approached statistical significance (p = .058); this suggests that future studies involving larger sample sizes and longer intervention periods could provide clearer evidence regarding the cognitive benefits of table tennis.

Notably, the greater improvement tendency observed in the table tennis group across both cognitive variables may suggest a potential facilitating role of table tennis in attention-related performance. This pattern, despite the absence of statistically significant interaction effects, indicates that table tennis may represent a promising activity for supporting cognitive skill development. Overall, although table tennis training was associated with greater improvement tendencies in cognitive variables, the findings do not provide sufficient statistical evidence to confirm a causal effect. Future studies are recommended to employ longer intervention periods, larger and more heterogeneous samples, and more controlled experimental designs in order to further investigate the cognitive effects of table tennis.

## 5. Suggestıons

To improve the quality of this study, several recommendations can be made based on the findings and limitations of the research.

First, future studies should consider extending the duration of the table tennis intervention beyond 8 weeks, as a longer training period may provide more reliable evidence regarding cognitive development outcomes. In addition, increasing the sample size and including participants from different age groups would enhance the generalizability of the results.

Second, it is recommended that future research conduct experimental studies across different sports branches in order to better compare the specific effects of table tennis on attention-related cognitive skills such as concentration and careful decision-making.

Third, the use of more diverse measurement tools, including standardized cognitive tests, questionnaires, and technological measurement devices, is suggested to increase the reliability and validity of the findings.

Moreover, incorporating mental development-oriented programs into table tennis lessons may further support cognitive improvement processes and provide a structured approach to enhancing attention skills.

Finally, qualitative methods such as interviews with participants who engage in table tennis training could provide deeper insights into whether improvements in concentration and decision-making skills transfer to daily life contexts. Considering the limitations of the present study, it is also important to note that external uncontrollable factors such as students’ academic course choices and participation in other sports activities may have influenced the observed outcomes. Future studies should aim to control or monitor these variables more strictly.

## Supporting information

S1 Dataset Anonymized SPSS dataset underlying the findings reported in the manuscript. (SAV)

S2 Dataset Anonymized Excel dataset underlying the findings reported in the manuscript. (XLSX)

## References

[1] Ferrandez C, Marsan T, Poulet Y, Rouch P, Thoreux P, Sauret C. Physiology, biomechanics and injuries in table tennis: a systematic review. Sci Sports. 2021;36(2):95–104. doi: 10.1016/j.scispo.2020.04.007

[2] Bischoff M, Zentgraf K, Pilgramm S, Stark R, Krüger B, Munzert J. Anticipating action effects recruits audiovisual movement representations in the ventral premotor cortex. Brain Cogn. 2014;92C:39–47. doi: 10.1016/j.bandc.2014.09.010 25463138

[3] Jeoung BJ. Relationships of exercise with frailty, depression, and cognitive function in older women. J Exerc Rehabil. 2014;10(5):291–4. doi: 10.12965/jer.140128 25426466 PMC4237844

[4] Genç Hİ, Hepsert S, Öktem T, Kiratli T, Yildirim Y, İlgin Ö. Orthorexia nervosa: an investigation in the context of personality, body image, and physical activity. Front Psychol. 2025;16:1672773. doi: 10.3389/fpsyg.2025.1672773 41280203 PMC12634581

[5] Tozlu A. Karar verme yaklaşımları üzerinde Herbert Simon hegemonyası. Sayıştay Dergisi. 2016;102:27–45.

[6] Sarikabak M, Hepsert S, Dönmez A, Baykara C, Cana H, Ayranci M, et al. Cognitive traces of life kinetic exercise in puberty sedentary subjects. PLoS One. 2025;20(5):e0322778. doi: 10.1371/journal.pone.0322778 40344562 PMC12064203

[7] Hepsert S, Dönmez A, Şakar İ, Cana H, Ramazanoğlu F. Satranç sporunun bilişsel esneklik ve problem çözme becerisi üzerindeki etkisi: Tanımlayıcı araştırma. Turkiye Klinikleri J Sports Sci. 2024;16(2).

[8] Fitts PM, Posner MI. Human Performance. Oxford (UK): Brooks/Cole; 1967.

[9] Liu M. The application of information technology in the reform of table tennis teaching in colleges. In: 2021 4th Int Conf on Information Systems and Computer Aided Education. 2021. pp. 1218–23.

[10] Awally AF, Suherman A, Subarjah H. The influence of teaching style and motivation level on increasing learning outcomes table tennis skills. Halaman Olahraga Nusantara. 2023;6(1):265–75.

[11] Peng Z, Xu L, Wang H, Song T, Shao Y, Liu Q, et al. The lateralization of spatial cognition in table tennis players: neuroplasticity in the dominant hemisphere. Brain Sci. 2022;12(12):1607. doi: 10.3390/brainsci12121607 36552067 PMC9775476

[12] Zhang S, Chen G, Wu Q, Li X. The interplay between table tennis skill development and sports performance: a comprehensive review. Pac Int J. 2023;6(3):150–6.

[13] Naderi A, Goli S, Shephard RJ, Degens H. Six-month table tennis training improves body composition, bone health and physical performance in untrained older men; a randomized controlled trial. Sci Sports. 2021;36(1):72.e1–72.e9. doi: 10.1016/j.scispo.2020.02.008

[14] Gül M, Çelik D. The effect of eight-weeks coordinatıon training on tennis and motor skills performance on 8-10 years children. Pak J Med Health Sci. 2021;15(6):1578–82. doi: 10.53350/pjmhs211561578

[15] Pradas F, Ara I, Toro V, Courel-Ibáñez J. Benefits of regular table tennis practice in body composition and physical fitness compared to physically active children aged 10-11 years. Int J Environ Res Public Health. 2021;18(6):2854. doi: 10.3390/ijerph18062854 33799620 PMC8000723

[16] Hertting K, Holmquist M, Parker J, Karlsson M, Sandéhn A. Ping pong health!: a table tennis intervention for improved health at the workplace. In: The Science and Practice of Racket Sports for Improved Performance and Health. Halmstad University; 2018. pp. 22–3.

[17] Yamasaki T. Benefits of table tennis for brain health maintenance and prevention of dementia. Encyclopedia. 2022;2(3):1577–89. doi: 10.3390/encyclopedia2030107

[18] Pan C-Y, Tsai C-L, Chu C-H, Sung M-C, Huang C-Y, Ma W-Y. Effects of physical exercise intervention on motor skills and executive functions in children with ADHD: a pilot study. J Atten Disord. 2019;23(4):384–97. doi: 10.1177/1087054715569282 25646023

[19] Fraenkel J, Wallen N, Hyun H. How to Design and Evaluate Research in Education. 10th ed. USA: McGraw-Hill Education; 1993.

[20] Faul F, Erdfelder E, Buchner A, Lang A-G. Statistical power analyses using G*Power 3.1: tests for correlation and regression analyses. Behav Res Methods. 2009;41(4):1149–60. doi: 10.3758/BRM.41.4.1149 19897823

[21] Cohen JD. Statistical power analysis for the behavioral sciences. 2nd ed. Lawrence Erlbaum Associates; 1988.

[22] Mann L, Burnett P, Radford M, Ford S. The Melbourne Decision Making Questionnaire: an instrument for measuring patterns for coping with decisional conflict. J Behav Decis Mak. 1997;10(1):1–19.

[23] Deniz ME. Investigation of the relation between decision making self-esteem, decision making style and problem solving skills of university students. Eurasian J Educ Res. 2004;4(15):23–35.

[24] Karagöz Y. Spss ve Amos uygulamalı nitel-nicel-karma bilimsel araştırma yöntemleri ve yayın etiği. 1 ed. İstanbul: Nobel Kitabevi; 2017.

[25] Brickencamp R. D2 Aufmerksamkeits-Belastungs Test: Handanweisung. Göttingen: Hofrere; 1981.

[26] Çağlar E, Koruç Z. D2 dikkat testinin sporcularda güvenirliği ve geçerliği. Spor Bil Derg. 2006;17(2):58–80.

[27] George D, Mallery P. IBM SPSS statistics 26 step by step: A simple guide and reference. 16 ed. New York, NY: Routledge; 2019.

[28] Işık O, Doğan I. Effects of bilateral or unilateral lower body resistance exercises on markers of skeletal muscle damage. Biomed J. 2018;41(6):364–8. 30709578 10.1016/j.bj.2018.10.003 PMC6361852

[29] Kawano MM, Mimura K, Kaneko M. The effect of table tennis practice on mental ability evaluated by Kana-Pick-out test. Int J Table Tennis Sci. 1992;1:57–62.

[30] Mori T, Sato T. Clinical brain sports medicine. Biomechanisms. 2004;17:1–8. doi: 10.3951/biomechanisms.17.1

[31] Visser A, Büchel D, Lehmann T, Baumeister J. Continuous table tennis is associated with processing in frontal brain areas: an EEG approach. Exp Brain Res. 2022;240(6):1899–909. doi: 10.1007/s00221-022-06366-y 35467129 PMC9142473

[32] Huang Q, Mao X, Shi J, Pan J, Li A. Enhanced cognitive inhibition in table tennis athletes: insights from color-word and spatial stroop tasks. Brain Sci. 2024;14(5):443. doi: 10.3390/brainsci14050443 38790422 PMC11117886

[33] Horníková H, Skala F, Zemková E. Sport-specific differences in key performance factors among handball, basketball and table tennis players. Int J Comput Sci Sport. 2023;22(1):31–41. doi: 10.2478/ijcss-2023-0003

[34] Haryanto J, Amra F. The relationship of concentration and eye-hand coordination with accuracy of backhand backspin serve in table tennis. Int J Technol Innov Humanit. 2020;1(1):51–6.

[35] Chen K-F, Chueh T-Y, Hung T-M. Differences in visuospatial cognition among table tennis players of different skill levels: an event-related potential study. PeerJ. 2024;12:e17295. doi: 10.7717/peerj.17295 38827290 PMC11144390

[36] Guo Z, Chang L. Unveiling the mental toughness and performance resilience of table tennis athletes. J Educ Educ Res. 2024;8(3):41–56.

